# Problematic Social Media Use and Lifestyle Behaviors in Adolescents: Cross-Sectional Questionnaire Study

**DOI:** 10.2196/46966

**Published:** 2023-12-28

**Authors:** Frank Hendrik Ardesch, Denise Dorothy van der Vegt, Jessica Christina Kiefte-de Jong

**Affiliations:** 1 Department of Public Health and Primary Care, Health Campus The Hague, Leiden University Medical Center, Leiden, Netherlands

**Keywords:** problematic social media use, lifestyle factors, adolescents, lifestyle behaviors, social media, addictive social media use, high school, users, risk factor, sociodemographic factors, addiction, internet, internet use, social media use

## Abstract

**Background:**

The use of social media by adolescents has increased considerably in the past decade. With this increase in social media use in our daily lives, there has been a rapidly expanding awareness of the potential unhealthy lifestyle-related health effects arising from excessive, maladaptive, or addictive social media use.

**Objective:**

This study aims to assess the association between adolescents’ social media use and health-related behaviors.

**Methods:**

We used a cross-sectional research approach and analyzed data from 96,919 adolescents at high schools throughout the Netherlands. A structured 43-item questionnaire was used to gather data on sociodemographics, dietary and lifestyle factors, and the degree of social media use based on the Compulsive Internet Use Scale. Logistic regression analyses were performed to assess the association between problematic social media use (PSMU) and lifestyle behaviors while adjusting for sociodemographic factors.

**Results:**

Of the 96,919 included adolescents, 7.4% (n=7022) were identified as at risk for PSMU. Furthermore, logistic regression results showed that adolescents who are at risk for PSMU were more likely to report alcohol consumption and smoking while simultaneously having significantly lower levels of health-promoting behavior such as healthy eating habits (eating fruits, vegetables, and breakfast regularly) and physical activity.

**Conclusions:**

This study confirms that adolescents at risk of PSMU were more likely to exhibit an unhealthy lifestyle. Being at risk for PSMU was a determinant of soft drug use, alcohol consumption, smoking, poor eating habits, and lower physical activity independent of the additional adjusted covariates including demographic variables and remaining lifestyle variables. Future research is needed to confirm this observation in an experimental setting.

## Introduction

Nowadays, social media is an important part of the daily life of adolescents. In today’s society, social media has been widely adopted, and adolescents have the highest rates of social media use of any age group [1]. As the time spent by adolescents online has almost doubled in the past decade, the widespread interest in how this might be affecting them led to the development of scientific evidence mapping potential associations between social media use and health outcomes [2,3].

Adolescence is a developmental stage in which parental influence decreases and the opinions of peers become more important in determining behavior [4,5]. Therefore, adolescence has been proposed to be an important time for the development of lasting health behaviors [6]. Following the behavior learning theory, individuals learn from one another via observation, imitation, and modeling [7]. Previous scientific evidence has shown that exposure to online behavior is a significant source of influence on adolescents’ health attitudes, intentions, and behaviors in which adolescents are motivated to fit into group identities and adopt the normative behaviors of their peers [4,8,9].

Social media provides clear benefits, of which social interaction is the most important [10]. The interactive nature can provide opportunities to engage with peers on issues and access support networks [4]. Other reasons for social media use are the gathering of information and entertainment purposes such as watching movies and listening to music [1,11,12]. Despite these benefits, the negative impacts of social media use have become increasingly apparent, in particular, excessive, maladaptive, or addictive use of social media, which is a condition also known by terms such as problematic social media use (PSMU) [13]. In general, PSMU can be defined as “Use of social media that creates physiological, social, school, and/or work difficulties in a person’s life” [14]. There are notable unhealthy lifestyle behaviors associated with PSMU among adolescents, such as physical inactivity, substance use, and poor dieting habits, and related to each other by confounding. Likewise, social media use is positively associated with sedentary behavior [11,15]. For adults, there is strong evidence linking sedentary behavior to a higher risk of overweight and obesity, cardiovascular disease, metabolic dysregulation, insufficient sleep, osteoporosis, and a reduction of psychosocial functioning [15,16]. Additionally, these negative health consequences are more likely to develop in adults who spent greater amounts of time sedentary in their youth [15,17]. These consequences highlight the importance of restricting sedentary time among young people and adolescents [15].

International concern for the well-being and health of adolescents has been growing after reports of increases in unhealthy lifestyle behaviors [18]. The literature showed both positive and negative associations between health behavior and social media whereby the overall conclusion states that the time spent on social media replaces time spent otherwise on health-related behaviors, such as physical activity or sleeping [1,3,5,11,12]. These findings are consistent with the displacement hypothesis, which declares that interactions through online relationships would displace the time allocated for offline activities, resulting in a disruption to one’s supposedly more valuable offline relationships [6,19]. However, most of the research on this topic has been conducted in Asian countries, and therefore, European results are of great interest as an addition to the current literature [14]. Furthermore, most studies did not account for other lifestyle factors that can confound the observed associations. Therefore, this study aimed to explore the health impacts related to a lifestyle of social media use among adolescents living in the Netherlands.

## Methods

### Data Source

The data used for the study were collected from the Public Health Monitor Youth 2015, which is a nationally representative observational cross-sectional survey of eighth and 10th grade adolescents in the Netherlands [20]. The Public Health Monitor Youth collected data through anonymous digital questionnaires in 596 high schools throughout the Netherlands. To obtain a nationally representative sample of schools and pupils, the survey used a random sampling design in a total of 25 municipal health care service regions. The size of samples varied per region. Schools were invited by letter to participate. Both parental consent and adolescents’ consent were obtained in advance of the questionnaire. Participation in this study was voluntary. Adolescents completed the questionnaire anonymously via the internet during class.

### Participants

A total of 596 schools were asked to participate. The school response rate to the survey was 63.1% (n=376), and nonresponse was mostly due to an overload of other surveys and fundamental objections. We derived the study sample from Public Health Monitor Youth surveys conducted in 2015 [20]. The data consisted of a pooled sample of 96,919 adolescents between the ages of 12 and 19 years who answered the questions on social media use and health behaviors.

### Ethical Considerations

Data collection procedures were approved (W19_148 # 19.183) by the ethical committee of The National Institute for Public Health and the Environment. Further details on the study design and methods are described elsewhere [20]. Following Dutch law (Wet medisch wetenschappelijk onderzoek met mensen), ethical review and approval were not required for the Public Health Monitor Youth as participants were not subjected to any intervention or treatment. Additionally, parents and children were informed by letters that by filling out the questionnaire, they consented to the anonymous use of data for research.

### Questionnaire

A questionnaire with 43 multiple-choice questions was used to elicit information regarding each adolescent’s social media use and lifestyle behavior (Multimedia Appendix 1). The questionnaire was divided into five parts. In the first part, adolescents were requested to respond to general and demographic questions (eg, sex, age, type of education, and origin). The second part covered the perceived physical, social, and mental health of the adolescents. The third part provided questions about their lifestyle behaviors. Questions on lifestyle behaviors included substance use (eg, “What types of substances have you ever used?”), alcohol consumption (eg, alcohol use per 4 wk and in a lifetime), smoking (eg, “Have you ever smoked?” and “How often do you smoke”), eating habits (breakfast habits and the number of days eating fruits or vegetables), and physical activity (number of days per week of physical activity for at least 1 h). In the fourth part, the adolescents were asked about their school life experiences (eg, functioning at school or bullying). The last part addressed questions related to adolescents’ social media use, gaming resilience, and sexuality. Questions related to social media referred to the use of messaging via smartphone, tablet, or PC (eg, WhatsApp or Snapchat); social network sites (eg, Facebook or Twitter); and forum sites. The frequency of social media use (“How often are you active on social media?”) was measured by a Likert-like scale with 6 response possibilities: never, <1 day per week, 1 day per week, 2-3 days per week, 4-5 days per week, or (almost) every day.

### Problematic Social Media Use

To measure the consequences of PSMU, the abbreviated version of the Compulsive Internet Use Scale from the Dutch research institute IVO was used [21]. The outcome variable, at risk for PSMU, was based on the following 7 items of the Compulsive Internet Use Scale: “How often do you find it difficult to quit social media?” “How often say others that you should spend less time on social media?” “How often would you rather use social media than spend time with others in real life?” “How often do you feel restless, stressed or annoyed when you can’t use social media?” “How often do you neglect your homework to use social media?” “How often are you using social media because you feel bad?” and “How often do you lack sleep through social media?” A Likert-like scale was used with 5 possible responses: never (0), seldom (1), sometimes (2), often (3), and very often (4). The mean values of the sum of the Likert scale responses were calculated, and a mean score of 0 to 2 was labeled as no or low risk of PSMU, and a total of 2 to 4 was labeled as at risk of PSMU. These cutoffs were based on the approach of Van Rooij et al [22] who found groups demarcating potential addiction and high-risk use for high engagement based on latent class analysis in the general population.

### Covariate Assessment

Covariates of interest were derived from the existing literature. All covariates were measured by the original questionnaire. The following covariates were considered important because of their relationship with PSMU: sex (male/female); age (continuous); family composition (two parents, stepfamily, co-parenting, single-parent household, or living with others or on its own); educational level (prevocational secondary education, senior general secondary education, or preuniversity education); soft drugs (never, ever, or recently used marijuana or hash); hard drugs (never, ever used, or recently using ecstasy, cocaine, psychedelic mushrooms, amphetamine, lysergic acid diethylamide, gamma hydroxybutyrate, heroin, or laughing gas); alcohol consumption (never, ever, or recently used alcohol); smoking (never smoked, ever smoked, or daily smoker); physical activity (weekly physical activity at a club or gym: yes/no); and eating breakfast (5 or more times a week: yes/no), fruits (5 or more times a week: yes/no), or vegetables (5 or more times a week: yes/no). For soft drugs, hard drugs, and alcohol, the “recently used” category indicates that someone used drugs or alcohol in the last 4 weeks.

### Statistical Analysis

The *P* value for trend was calculated by using a linear regression model. Results of the multiple logistic regression models to analyze the association between PSMU and individual lifestyle factors were presented in an unadjusted model (model 1); a model adjusted for sex, age, and educational level (model 2); and a model between the risk of PSMU and lifestyle behaviors, corrected for sex, age, educational level, family composition, mutual adjustment for substance use (soft and hard drugs), alcohol consumption, smoking, physical activity, and eating habits (fruit, vegetable, and breakfast intake; model 3). The associations were reported as odds ratios (ORs) with 95% CIs. *P* values <.05 were considered to be statistically significant. Missing values were imputed by using the Markov chain Monte Carlo method. After the imputation procedure, the effect estimates were pooled. The pooled estimates were used to perform the analyses in this study. All analyses were performed by using the statistical software SPSS version 26 (IBM Corp).

## Results

### Study Population Characteristics

Table 1 presents the characteristics of the study sample over two groups based on the risk for PSMU. The study sample consisted of adolescents who answered questions on social media use and health behaviors (N=96,919) in which 89,710 (92.6%) of the adolescents were not at risk or were at low risk for PSMU, while 7209 (7.4%) were at risk for PSMU. The at-risk PSMU group consisted of a higher number of female adolescents 4749 (65.9%). Both groups had approximately equal mean ages (14.29, SD 1.25 years vs 14.48, SD 1.22 years), ranging from 12 to 19 years. Preuniversity education was more common among low-risk users (n=19,151, 21.4%) than in the at-risk PSMU group (n=856, 11.9%), and the majority of both groups attended prevocational school or senior general secondary education. Lifestyle behaviors such as substance use, alcohol consumption, and smoking were more commonly observed among the at-risk PSMU group. No-risk or low-risk users skipped breakfast, fruit, and vegetable intake less often compared to the at-risk PSMU group.

**Table 1.** Baseline characteristics of included adolescents (N=96,919).

			No or low-risk problematic social media use	At-risk problematic social media use	*P* value
Total, n (%)			89,710 (92.6)	7209 (7.4)	N/A^a^
Sex (female), n (%)			43,292 (48.3)	4749 (65.9)	<.001
Age (years), mean (SD)			14.29 (1.25)	14.48 (1.22)	<.001
**Family composition, n (%)**					<.001
	Two parents		68,537 (76.4)	4877 (67.7)	
	Stepfamily		5801 (6.5)	647 (9.0)	
	Co-parents		5838 (6.5)	518 (7.2)	
	Single parent		8573 (9.6)	956 (13.3)	
	With others or on its own		961 (1.0)	211 (2.9)	
**Educational level, n (%)**					<.001
	Prevocational secondary education		45,760 (51)	4622 (64.1)	
	Senior general secondary education		24,799 (27.6)	1731 (24.0)	
	Preuniversity education		19,151 (21.4)	856 (11.9)	
**Substance use, n (%)**					
	**Soft drugs**				<.001
		Never used	81,190 (90.5)	5601 (77.7)	
		Recently used	4248 (4.7)	917 (12.7)	
		Ever used	4272 (4.8)	693 (9.6)	
	**Hard drugs**				<.001
		Never used	88,193 (98.3)	6819 (94.6)	
		Recently used	546 (0.6)	149 (2.1)	
		Ever used	972 (1.1)	241 (3.3)	
**Alcohol use, n (%)**					<.001
	Never used		59,433 (66.3)	3096 (42.9)	
	Recently used		23,510 (26.2)	3398 (47.1)	
	Ever used		6767 (7.5)	715 (10.0)	
**Smoking, n (%)**					<.001
	Never smoked		77,081 (85.9)	4778 (66.3)	
	Ever smoked		8749 (9.8)	1563 (21.7)	
	Daily smoker		3881 (4.3)	868 (12.0)	
**Physical activity, n (%)**					<.001
	Weekly, yes		69,794 (78.1)	4968 (68.9)	
**Eating habits, n (%)**					
	Breakfast ≥5 d/wk, yes		76,194 (84.9)	4625 (64.2)	<.001
	Fruit ≥5 d/wk, yes		45,160 (50.3)	2661 (36.9)	<.001
	Vegetables ≥5 d/wk, yes		72,187 (80.5)	4937 (68.5)	<.001

a N/A: not applicable.

### PSMU and Lifestyle Behaviors

The association between being at risk for PSMU and various lifestyle variables is shown in Table 2. Model 1 demonstrates that, without any adjustments, being at risk for PSMU was positively associated with soft drugs (recently used: OR 3.12, 95% CI 2.90-2.35; ever used: OR 3.12, 95% CI 2.90-2.35) and hard drugs (recently used: OR 3.53, 95% CI 2.88-4.33; ever used: OR 3.20, 95% CI 2.67-3.84), alcohol consumption (recently used: OR 2.77, 95% CI 2.71-2.83; ever used: OR 2.03, 95% CI 1.96-2.10), and smoking (recently used: OR 2.88, 95% CI 2.71-3.07; ever used: OR 3.61, 95% CI 3.50-3.73), and negatively associated with physical activity (OR 0.63, 95% CI 0.62-0.65) and fruit (OR 0.58, 95% CI 0.57-0.59), vegetable (OR 0.53, 95% CI 0.52-0.54), and breakfast intake (OR 0.32, 95% CI 0.31-0.33). After adjusting for sex, age, and educational level (model 2), the model showed similar significant results. Furthermore, model 3 revealed that after adjusting for sex, age, educational level, household composition, and the remaining lifestyle behaviors, only the association with hard drugs became nonsignificant (recently used: OR 1.12, 95% CI 0.90-1.39; ever used: OR 1.09, 95% CI 0.87-1.37) when compared to the other models.

**Table 2.** Logistic regression results showing the association between the risk of problematic social media use and lifestyle behaviors among the adolescents.

Outcome variable		Model 1^a^, OR^b^ (95% CI)	Model 2^c^, OR (95% CI)	Model 3^d^, OR (95% CI)
**Soft drugs**				
	Never used	1.00 (reference)	1.00 (reference)	1.00 (reference)
	Recently used	3.12 (2.90-3.37)^e^	3.32 (3.06-3.60)^e^	1.43 (1.29-1.59)^e^
	Ever used	2.35 (2.16-2.56)^e^	2.32 (2.12-2.54)^e^	1.19 (1.07-1.32)^e^
**Hard drugs**				
	Never used	1.00 (reference)	1.00 (reference)	1.00 (reference)
	Recently used	3.53 (2.88-4.33)^e^	3.30 (2.66-4.09)^e^	1.12 (0.90-1.39)
	Ever used	3.20 (2.67-3.84)^e^	2.72 (2.24-3.29)^e^	1.09 (0.87-1.37)
**Alcohol use**				
	Never used	1.00 (reference)	1.00 (reference)	1.00 (reference)
	Recently used	2.77 (2.71-2.83)^e^	3.11 (3.03-3.18)^e^	2.26 (2.10-2.43)^e^
	Ever used	2.03 (1.96-2.10)^e^	2.20 (2.12-2.28)^e^	1.80 (1.64-1.98)^e^
**Smoking**				
	Never used	1.00 (reference)	1.00 (reference)	1.00 (reference)
	Ever used	2.88 (2.71-3.07)^e^	2.73 (2.66-2.80)^e^	1.51 (1.47-1.56)^e^
	Daily use	3.61 (3.50-3.73)^e^	3.37 (2.25-3.49)^e^	1.32 (1.26-1.38)^e^
**Weekly physical activity**				
	No	1.00 (reference)	1.00 (reference)	1.00 (reference)
	Yes	0.63 (0.62-0.65)^e^	0.74 (0.72-0.75)^e^	0.84 (0.82-0.86)^e^
**Fruit intake**				
	<5 d/wk	1.00 (reference)	1.00 (reference)	1.00 (reference)
	≥5 d/wk	0.58 (0.57-0.59)^e^	0.59 (0.58-0.60)^e^	0.73 (0.72-0.75)^e^
**Vegetable intake**				
	<5 d/wk	1.00 (reference)	1.00 (reference)	1.00 (reference)
	≥5 d/wk	0.53 (0.52-0.54)^e^	0.55 (0.54-0.56)^e^	0.67 (0.65-0.69)^e^
**Eating breakfast**				
	<5 d/wk	1.00 (reference)	1.00 (reference)	1.00 (reference)
	≥5 d/wk	0.32 (0.31-0.33)^e^	0.38 (0.37-0.39)^e^	0.52 (0.50-0.53)^e^

a Model 1: unadjusted.

b OR: odds ratio.

c Model 2: adjusted for sex, age, and educational level.

d Model 3: adjusted for sex; age; educational level; household composition; soft drugs; hard drugs; alcohol; smoking; physical activity; and fruit, vegetable, and breakfast intake.

e *P*<.001

## Discussion

This study showed that adolescents who are at risk of PSMU were more likely to exhibit an unhealthy lifestyle. Being at risk for PSMU was positively associated with substance use, alcohol consumption, and smoking, and negatively associated with physical activity and eating breakfast, vegetables, and fruits regularly, independent of the additional adjusted covariates including demographic variables and remaining lifestyle variables.

In this survey, the prevalence of PSMU was 7.4%, which is mostly in line with previously conducted studies [23-25]. The international variations of PSMU prevalence rates are reported on in a recent study by Cheng et al [26]. In Europe and the United States, prevalence rates ranged from 7.9% to 25.2% among adolescents, and prevalence rates in the Middle East and Africa ranged from 17.3% to 23.6% [23]. Moreover, the highest variation in prevalence among adolescents was observed in Asian studies, with a reported prevalence between 8.1% and 50.9% [23]. Nevertheless, the direct comparison of these studies is complicated due to the different diagnostic criteria and methodologies used (eg, the lack of a consensual definition of PSMU) [23,27]. This study found an independent association of social media with alcohol consumption and less physical activity, which is comparable with the previous Dutch study by Busch et al [28]. They investigated the relationship between screen time (including excessive internet, TV, and video gaming) and several health-related behaviors (eg, soft drugs, alcohol use, smoking, unsafe sex, skipping school, bullying, poor nutritional behavior, and physical activity) in adolescents. The results demonstrated that screen time was independently associated with alcohol consumption, bullying, and less physical activity. However, this study did not account for the mutual relation between health behaviors and only adjusted the analyses for demographic characteristics.

Screen-based sedentary behaviors have been recognized as a significant contributor to negative health indicators in various aspects of adolescents [16,29]. Physical consequences consisted of being overweight and having risk factors for cardiovascular diseases (eg, obesity, hypertension, and high-density lipoprotein dysfunction) due to a lack of physical activity and passive food consumption [16,30,31]. Adolescent screen time and skipping breakfast regularly are associated with a higher calorie intake [32]. Eating in front of a screen and not having a regular breakfast routine can lead to excessive snacking and poor food choices [33]. This results in a higher calorie intake, which can contribute to weight gain and other health problems. Furthermore, sleep quality is affected by exposure to bright and blue lights emitted by digital devices that may suppress melatonin production and cause circadian disruption [16,34]. Another mechanism that affects sleep is chronic sympathetic arousal. Psychophysiological arousal may increase due to playing video games, which leads to sympathetic dysregulation [16,34]. As a result, pre-bedtime relaxation may be impeded, which leads to delayed and shortened sleep [16,35]. At the same time, chronic sympathetic arousal is also associated with metabolic dysregulation, including lower levels of cortisol and insulin resistance [16]. Lastly, a study by French et al [36] observed that outdoor activity stimulates the release of dopamine from the retina. This release of dopamine suppresses the development of myopia. Hence, adolescents who spend more time inside are more likely to become myopic.

Neurophysiological issues can accompany PSMU. One of these issues is a low level of support, resulting in decreased social coping, for example, less social support and attachment with family and peers [16,37,38]. This decrease in social coping comes at the expense of face-to-face contact, which in turn is strongly associated with positive well-being and life satisfaction [16,38]. All of these components together increase the risk for depression, isolation, and loneliness, and may further maintain addictive behavior [16,37]. Likewise, neuroanatomical changes may occur, including decreased impulse control and dysfunctional decision-making and emotional processing, and can involve craving behavior and maintain addictive behavior [16]. Furthermore, it is known from recent studies that several health-related behaviors have a clustered profile. These behaviors influence each other instead of acting independently on one’s health [39]. This clustered profile is accompanied by a synergetic effect, which means that certain behaviors increase the likelihood of being involved in other risk behaviors [40]. Consequently, due to this covariance, the risk of disease is higher with clustered behaviors compared to nonclustered behaviors. This increase can be explained by the “Gateway” hypothesis that, on top of the health risks that come with certain risk behaviors, someone’s mindset and decision-making abilities are affected by partaking in other risk-taking behaviors [39,41,42]. Empirical support for this theory has been found. For example, alcohol users are more likely to take part in smoking than nonalcohol drinkers [39,41]. Another explanatory hypothesis for the co-occurrence of risk behaviors during adolescence is the “Problem Behavior Theory,” which suggests that partaking in “problem behavior” in early adolescence is enacted as a means of demonstrating maturity and independence and repudiating conventionality [42]. This theory has been empirically supported by a few studies that analyzed both common risk and protective factors for risk behaviors [42,43].

This study was strengthened by the fact that this study was conducted with nearly 100,000 adolescents in the Netherlands, using one of the largest surveys ever conducted in terms of the number of samples of epidemiological research into PSMU among adolescents [44]. Besides the large sample size, the participating schools were randomly selected nationwide. This random selection supports the assumption that this sample is representative of the entire Dutch population. Analyses were also strengthened by not only adjusting for potential confounding factors (sex, age, and socioeconomic status) but also mutually adjusting for other lifestyle factors as unhealthy behaviors cluster together [45]. A limitation of this study was that the data was collected as a cross-sectional study. For this reason, it is not possible to determine whether significant associations between PSMU and the presumed outcomes such as an unhealthy lifestyle are causal or whether adolescents with an unhealthy lifestyle are more likely to engage in PSMU; additional longitudinal data assessments with multiple measures of social media addiction are needed. Additionally, social desirability may have influenced the results, as the data was collected using a self-reported questionnaire. However, since the questionnaire was anonymous and not linked to adolescents’ health records, bias due to social desirability was more likely to be diminished. Another shortcoming is the missing data about mental health. The psychiatric profile of the adolescents was not assessed in this study and could be an important factor according to recent research [46]. Therefore, it is possible that mental health problems such as depression have gone unnoticed and may have biased the outcome results, assuming that adolescents with mental issues are less likely to make healthy lifestyle choices. Furthermore, the questionnaire has not been validated. Therefore, differential misclassification could influence the results. Lastly, it can be asserted that, over the past 8 years, social media has undergone transformations that make contextual alignment between 2015 and 2023 somewhat challenging. Nevertheless, we intentionally opted to use pre–COVID-19 pandemic data due to its reduced bias. Subsequent research endeavors should aim to replicate our findings using the same methodology to validate our outcomes.

Concerns about PSMU have increased since the prevalence increased due to the COVID-19 pandemic [47-49]. Given its impact on health, it is important to develop interventions to reduce PSMU. Both pharmacotherapeutic and psychological interventions have been studied to reduce PSMU [50]. Pharmacotherapeutic interventions have mainly paid attention to dopamine regulators and selective serotonin reuptake inhibitors since these medications have proven effective for other psychological conditions such as attention-deficit/hyperactivity disorder, substance use disorder, and obsessive-compulsive disorder [51,52]. A recent meta-analysis by Kim and Noh [53] showed that cognitive behavioral therapy, family-based interventions, and counseling programs can reduce the severity of PSMU [53]. However, evidence on which intervention is most effective in reducing the severity of PSMU and its subsequent impact on health and health behavior is limited. Given the nearly universal accessibility of social media, public health authorities have the opportunity to disseminate messages to adolescents in an innovative way using social media to promote healthy decision-making and thereby a healthy lifestyle. Nowadays, there is considerable interest in digital interventions for behavior change that seem cost-effective [54]. Despite the wide use of some health apps or devices, only a few health apps contain evidence-based behavioral change strategies or theoretical frameworks, or are based on clinical guidelines, and most of them ignore the totality of health behaviors including PSMU [55,56]. Therefore, the validation of the effectiveness of these digital interventions requires future research to address both the challenge of PSMU as well as other health behaviors.

Social media use is currently one of the most popular activities in today’s world, which has further increased after the COVID-19 pandemic, with adolescents being more likely to develop PSMU. While social media use is not inherently negative, offering several benefits such as social connection and interaction, excessive and uncontrolled social media use can be accompanied by negative health-related behaviors. This study confirmed that adolescents who are at risk of PSMU were more likely to exhibit an unhealthy lifestyle. Being at risk for PSMU was an independent risk factor for substance use, alcohol consumption, smoking, poor eating habits, and physical activity independent of the additional adjusted covariates including demographic variables and remaining lifestyle variables. To develop effective social media interventions, public health must prioritize studying the clustering of multiple health-related behaviors.

## Supplementary material

10.2196/46966 Multimedia Appendix 1 Basisvragenlijst Gezondheidsmonitor Jeugd 2015.

## Abbreviations

OR: odds ratio
PSMU: problematic social media use

## References

[1] Goodyear VA, Armour KM, Wood H (2018). Young people and their engagement with health-related social media: new perspectives. Sport Educ Soc.

[2] Orben A, Przybylski AK (2019). The association between adolescent well-being and digital technology use. Nat Hum Behav.

[3] Orben A (2020). Screens and social media: a narrative review of reviews and key studies. Soc Psychiatry Psychiatr Epidemiol.

[4] Montgomery SC, Donnelly M, Bhatnagar P, Carlin A, Kee F, Hunter RF (2020). Peer social network processes and adolescent health behaviors: a systematic review. Prev Med.

[5] Vannucci A, Simpson EG, Gagnon S, Ohannessian CM (2020). Social media use and risky behaviors in adolescents: a meta-analysis. J Adolesc.

[6] Vaterlaus JM, Patten EV, Roche C, Young JA (2015). Gettinghealthy: the perceived influence of social media on young adult health behaviors. Comput Hum Behav.

[7] Telzer EH, van Hoorn J, Rogers CR, Do KT (2018). Social influence on positive youth development: a developmental neuroscience perspective. Adv Child Dev Behav.

[8] O’Reilly M (2020). Social media and adolescent mental health: the good, the bad and the ugly. J Ment Health.

[9] Stok FM, de Vet E, de Ridder DTD, de Wit JBF (2016). The potential of peer social norms to shape food intake in adolescents and young adults: a systematic review of effects and moderators. Health Psychol Rev.

[10] Gonzales AL, Hancock JT (Jan-Feb 2011). Mirror, mirror on my Facebook wall: effects of exposure to Facebook on self-esteem. Cyberpsychol Behav Soc Netw.

[11] Sampasa-Kanyinga H, Chaput JP, Hamilton HA (2019). Social media use, school connectedness, and academic performance among adolescents. J Prim Prev.

[12] Shimoga SV, Erlyana E, Rebello V (2019). Associations of social media use with physical activity and sleep adequacy among adolescents: cross-sectional survey. J Med Internet Res.

[13] Kamal NN, Mosallem FH (2013). Determinants of problematic internet use among El-Minia high school students, Egypt. Int J Prev Med.

[14] Wang H, Zhou X, Lu C, Wu J, Deng X, Hong L (2011). Problematic internet use in high school students in Guangdong Province, China. PLoS ONE.

[15] Hardy LL, Denney-Wilson E, Thrift AP, Okely AD, Baur LA (2010). Screen time and metabolic risk factors among adolescents. Arch Pediatr Adolesc Med.

[16] Lissak G (2018). Adverse physiological and psychological effects of screen time on children and adolescents: literature review and case study. Environ Res.

[17] Hancox RJ, Milne BJ, Poulton R (2004). Association between child and adolescent television viewing and adult health: a longitudinal birth cohort study. Lancet.

[18] Goodyear VA, Armour KM, Wood H (2018). The Impact of Social Media on Young People’s Health and Wellbeing: Evidence, Guidelines and Actions.

[19] Nie NH, Hillygus DS (2002). The impact of Internet use on sociability: time-diary findings. IT Soc.

[20] Gezondheidsmonitor Jeugd 2015. De Gezondheidsmonitors.

[21] Meerkerk GJ, Van Den Eijnden R, Vermulst AA, Garretsen HFL (2009). The Compulsive Internet Use Scale (CIUS): some psychometric properties. Cyberpsychol Behav.

[22] Van Rooij AJ, Schoenmakers TM, Vermulst AA, Van den Eijnden R, Van de Mheen D (2011). Online video game addiction: identification of addicted adolescent gamers. Addiction.

[23] Hassan T, Alam MM, Wahab A, Hawlader MD (2020). Prevalence and associated factors of internet addiction among young adults in Bangladesh. J Egypt Public Health Assoc.

[24] Vigna-Taglianti F, Brambilla R, Priotto B, Angelino R, Cuomo G, Diecidue R (2017). Problematic internet use among high school students: prevalence, associated factors and gender differences. Psychiatry Res.

[25] Mihara S, Osaki Y, Nakayama H (2016). Internet use and problematic internet use among adolescents in Japan: a nationwide representative survey. Addict Behav Rep.

[26] Cheng C, Lau YC, Chan L, Luk JW (2021). Prevalence of social media addiction across 32 nations: meta-analysis with subgroup analysis of classification schemes and cultural values. Addict Behav.

[27] Liu TC, Desai RA, Krishnan-Sarin S, Cavallo DA, Potenza MN (2011). Problematic internet use and health in adolescents: data from a high school survey in Connecticut. J Clin Psychiatry.

[28] Busch V, Manders LA, de Leeuw JRJ (2013). Screen time associated with health behaviors and outcomes in adolescents. Am J Health Behav.

[29] Musa S, Elyamani R, Dergaa I (2022). COVID-19 and screen-based sedentary behaviour: systematic review of digital screen time and metabolic syndrome in adolescents. PLoS One.

[30] Merghani A, Malhotra A, Sharma S (2016). The U-shaped relationship between exercise and cardiac morbidity. Trends Cardiovasc Med.

[31] Mihrshahi S, Drayton BA, Bauman AE, Hardy LL (2017). Associations between childhood overweight, obesity, abdominal obesity and obesogenic behaviors and practices in Australian homes. BMC Public Health.

[32] Rocha LL, Gratão LHA, Carmo AS (2021). School type, eating habits, and screen time are associated with ultra-processed food consumption among Brazilian adolescents. J Acad Nutr Diet.

[33] Tambalis KD, Panagiotakos DB, Psarra G, Sidossis LS (2020). Screen time and its effect on dietary habits and lifestyle among schoolchildren. Cent Eur J Public Health.

[34] Cheung CHM, Bedford R, Saez De Urabain IR, Karmiloff-Smith A, Smith TJ (2017). Daily touchscreen use in infants and toddlers is associated with reduced sleep and delayed sleep onset. Sci Rep.

[35] Magee CA, Lee JK, Vella SA (2014). Bidirectional relationships between sleep duration and screen time in early childhood. JAMA Pediatr.

[36] French AN, Ashby RS, Morgan IG, Rose KA (2013). Time outdoors and the prevention of myopia. Exp Eye Res.

[37] Schou Andreassen C, Billieux J, Griffiths MD (2016). The relationship between addictive use of social media and video games and symptoms of psychiatric disorders: a large-scale cross-sectional study. Psychol Addict Behav.

[38] Pea R, Nass C, Meheula L (2012). Media use, face-to-face communication, media multitasking, and social well-being among 8- to 12-year-old girls. Dev Psychol.

[39] Busch V, Van Stel HF, Schrijvers AJP, de Leeuw JRJ (2013). Clustering of health-related behaviors, health outcomes and demographics in Dutch adolescents: a cross-sectional study. BMC Public Health.

[40] Pronk NP, Anderson LH, Crain AL (2004). Meeting recommendations for multiple healthy lifestyle factors. prevalence, clustering, and predictors among adolescent, adult, and senior health plan members. Am J Prev Med.

[41] Huang DYC, Lanza HI, Murphy DA, Hser YI (2012). Parallel development of risk behaviors in adolescence: potential pathways to co-occurrence. Int J Behav Dev.

[42] Hale DR, Viner RM (2016). The correlates and course of multiple health risk behaviour in adolescence. BMC Public Health.

[43] Jackson C, Sweeting H, Haw S (2012). Clustering of substance use and sexual risk behaviour in adolescence: analysis of two cohort studies. BMJ Open.

[44] Kuss DJ, Griffiths MD, Karila L, Billieux J (2014). Internet addiction: a systematic review of epidemiological research for the last decade. Curr Pharm Des.

[45] Geraets AFJ, Heinz A (2023). The associations of dietary habits with health, well-being, and behavior in adolescents: a cluster analysis. Child Care Health Dev.

[46] Orben A, Przybylski AK, Blakemore SJ, Kievit RA (2022). Windows of developmental sensitivity to social media. Nat Commun.

[47] Oka T, Hamamura T, Miyake Y (2021). Prevalence and risk factors of internet gaming disorder and problematic internet use before and during the COVID-19 pandemic: a large online survey of Japanese adults. J Psychiatr Res.

[48] Islam MS, Sujan MSH, Tasnim R (2020). Problematic internet use among young and adult population in Bangladesh: correlates with lifestyle and online activities during the COVID-19 pandemic. Addict Behav Rep.

[49] Nilsson A, Rosendahl I, Jayaram-Lindström N (2022). Gaming and social media use among adolescents in the midst of the COVID-19 pandemic. Nordisk Alkohol Nark.

[50] Kuss DJ, Lopez-Fernandez O (2016). Internet addiction and problematic internet use: a systematic review of clinical research. World J Psychiatry.

[51] Zajac K, Ginley MK, Chang R, Petry NM (2017). Treatments for internet gaming disorder and internet addiction: a systematic review. Psychol Addict Behav.

[52] Saquib J (Mar-Apr 2020). Internet addiction among Saudi Arabian youth. Int J Health Sci (Qassim).

[53] Kim S, Noh D (2019). The current status of psychological intervention research for internet addiction and internet gaming disorder. Issues Ment Health Nurs.

[54] Rose T, Barker M, Maria Jacob C (2017). A systematic review of digital interventions for improving the diet and physical activity behaviors of adolescents. J Adolesc Health.

[55] Arigo D, Jake-Schoffman DE, Wolin K, Beckjord E, Hekler EB, Pagoto SL (2019). The history and future of digital health in the field of behavioral medicine. J Behav Med.

[56] Nikolaou CK, Lean MEJ (2017). Mobile applications for obesity and weight management: current market characteristics. Int J Obes (Lond).

