# Supplementary material for: Problematic Social Media Use and Lifestyle Behaviors in Adolescents: Cross-Sectional Questionnaire Study
Source: JMIR Pediatr Parent. 2023 Dec 28;6:e46966. doi: 10.2196/46966 (PMC10775956; doi:10.2196/46966)
Supplement: Multimedia Appendix 1 [file pediatrics-v6-e46966-s001.docx]

Appendix 1: Basisvragenlijst Gezondheidsmonitor Jeugd 2015

*Versie 1 september 2015 - DEF*

Beste leerling,

Belangrijke **tips** voordat je de vragenlijst invult:

- Er zijn geen goede of foute antwoorden. Het gaat om jouw mening en jouw ervaringen.
- Het is belangrijk dat je alle vragen beantwoordt, ook al vind je het soms moeilijk om een antwoord te geven.
- Dit onderzoek is vertrouwelijk. Dat betekent dat aan jouw antwoorden geen naam of adres gekoppeld kan worden. Niemand komt dus te weten wat jij hebt ingevuld.

## Veel succes!

**Vraag 1. In welke klas/leerjaar zit je?**

❑ 1

❑ 2

❑ 3

❑ 4

❑ 5

❑ 6

## Vraag 2. Welk soort onderwijs volg je?

*Je mag meer dan één antwoord geven.*

❑ Vmbo-b (basis)

❑ Vmbo-k (kader)

❑ Vmbo-g (gemengd)

❑ Vmbo-t (theoretisch, mavo)

❑ Havo

❑ Vwo (atheneum, gymnasium)

❑ Ander soort onderwijs

## Vraag 3. Hoe oud ben je?

❑ 12 jaar of jonger

❑ 13 jaar

❑ 14 jaar

❑ 15 jaar

❑ 16 jaar

❑ 17 jaar

❑ 18 jaar of ouder

## Vraag 4. Ben je een jongen of een meisje?

❑ Jongen

❑ Meisje

## Vraag 5. Wat zijn de 4 cijfers van de postcode van het adres waar je woont?

**Vraag 6. In welk land ben je geboren?**

❑ Nederland

❑ Marokko

❑ Turkije

❑ Suriname

❑ (Voormalige) Nederlandse Antillen of Aruba

❑ Duitsland

❑ België

❑ Indonesië

❑ Ander land, namelijk [in opklapscherm keuze uit alle landen]

## Vraag 7. In welk land is je moeder geboren?

❑ Nederland

❑ Marokko

❑ Turkije

❑ Suriname

❑ (Voormalige) Nederlandse Antillen of Aruba

❑ Duitsland

❑ België

❑ Indonesië

❑ Ander land, namelijk [in opklapscherm keuze uit alle landen]

## Vraag 8. In welk land is je vader geboren?

❑ Nederland

❑ Marokko

❑ Turkije

❑ Suriname

❑ (Voormalige) Nederlandse Antillen of Aruba

❑ Duitsland

❑ België

❑ Indonesië

❑ Ander land, namelijk [in opklapscherm keuze uit alle landen]

## Vraag 9. Bij wie woon je de meeste dagen van de week?

*Je mag één antwoord geven.*

❑ Bij mijn ouders (samen)

❑ Ongeveer de helft van de tijd bij mijn ene ouder en de helft van de tijd bij mijn andere ouder

❑ Bij mijn moeder en haar partner

❑ Bij mijn vader en zijn partner

❑ Alleen bij mijn moeder

❑ Alleen bij mijn vader

❑ Bij anderen (bijv. pleegouders, andere familie, internaat)

❑ Ik woon op mezelf

## Vraag 10. Hoe is over het algemeen je gezondheid?

❑ Zeer goed

❑ Goed

❑ Gaat wel

❑ Slecht

❑ Zeer slecht

## Vraag 11. Wil je alsjeblieft bij iedere vraag een kruisje zetten in het vakje voor "Niet waar", "Een beetje waar" of "Zeker waar". Het is belangrijk dat je alle vragen zo goed mogelijk beantwoordt, ook als je niet helemaal zeker bent of als je de vraag raar vindt.

**Wil je alsjeblieft bij je antwoorden denken hoe dat bij jou de laatste 6 maanden is geweest.**

|  | Niet  waar | Een beetje  waar | Zeker  waar |
| --- | --- | --- | --- |
| Ik probeer aardig te zijn tegen anderen. Ik houd rekening met hun gevoelens | ❑ | ❑ | ❑ |
| Ik ben rusteloos, ik kan niet lang stil zitten | ❑ | ❑ | ❑ |
| Ik heb vaak hoofdpijn, buikpijn, of ik ben misselijk | ❑ | ❑ | ❑ |
| Ik deel makkelijk met anderen (snoep, speelgoed, potloden, etc.) | ❑ | ❑ | ❑ |
| Ik word erg boos en ben vaak driftig | ❑ | ❑ | ❑ |
| Ik ben nogal op mijzelf. Ik speel meestal alleen of bemoei mij niet met anderen | ❑ | ❑ | ❑ |
| Ik doe meestal wat me wordt opgedragen | ❑ | ❑ | ❑ |
| Ik pieker veel | ❑ | ❑ | ❑ |
| Ik help iemand die zich heeft bezeerd, van streek is of zich ziek voelt | ❑ | ❑ | ❑ |
| Ik zit constant te wiebelen of te friemelen | ❑ | ❑ | ❑ |
| Ik heb minstens één goede vriend of vriendin | ❑ | ❑ | ❑ |
| Ik vecht vaak. Het lukt mij andere mensen te laten doen wat ik wil | ❑ | ❑ | ❑ |
| Ik ben vaak ongelukkig, in de put of in tranen | ❑ | ❑ | ❑ |
| Andere jongeren van mijn leeftijd vinden mij over het algemeen aardig | ❑ | ❑ | ❑ |
| Ik ben snel afgeleid, ik vind het moeilijk om me te concentreren | ❑ | ❑ | ❑ |
| Ik ben zenuwachtig in nieuwe situaties. Ik verlies makkelijk mijn zelfvertrouwen | ❑ | ❑ | ❑ |
| Ik ben aardig tegen jongere kinderen | ❑ | ❑ | ❑ |
| Ik word er vaak van beschuldigd dat ik lieg of bedrieg | ❑ | ❑ | ❑ |
| Andere kinderen of jongeren pesten of treiteren mij | ❑ | ❑ | ❑ |
| Ik bied vaak anderen aan hen te helpen (ouders, leerkrachten, kinderen) | ❑ | ❑ | ❑ |
| Ik denk na voor ik iets doe | ❑ | ❑ | ❑ |
| Ik neem dingen weg die niet van mij zijn thuis, op school of op andere plaatsen | ❑ | ❑ | ❑ |
| Ik kan beter met volwassenen opschieten dan met jongeren van mijn leeftijd | ❑ | ❑ | ❑ |
| Ik ben voor heel veel dingen bang, ik ben snel angstig | ❑ | ❑ | ❑ |
| Ik maak af waar ik mee bezig ben. Ik kan mijn aandacht er goed bij houden | ❑ | ❑ | ❑ |

De onderstaande uitspraken gaan over opkomen voor jezelf en het maken van keuzes (zoals over pesten, kleding, uitgaan, roken, alcoholgebruik, seksualiteit).

## Vraag 12. Geef aan in hoeverre je het eens bent met onderstaande uitspraken.

*Kruis op iedere regel één vakje aan.*

| Helemaal mee eens | | Mee eens | Soms wel, soms niet | Mee oneens | Helemaal mee oneens |
| --- | --- | --- | --- | --- | --- |
| Ik weet wat ik wel en niet wil | ❑ | ❑ | ❑ | ❑ | ❑ |
| Ik zeg het als iemand iets doet wat ik vervelend vind | ❑ | ❑ | ❑ | ❑ | ❑ |
| Ik laat mij makkelijk overhalen om dingen te doen die ik niet wil | ❑ | ❑ | ❑ | ❑ | ❑ |
| Ik kan heel goed nee zeggen tegen mijn vrienden | ❑ | ❑ | ❑ | ❑ | ❑ |
| Ik vind het moeilijk om hulp te vragen als iemand mij lastig valt | ❑ | ❑ | ❑ | ❑ | ❑ |
| Ik doe alleen maar dingen die ik zelf echt wil | ❑ | ❑ | ❑ | ❑ | ❑ |
| Ik kom voor mezelf op als iemand mij uitscheldt, beledigt of bedreigt | ❑ | ❑ | ❑ | ❑ | ❑ |
| Als mijn vrienden iets doen wat ik  eigenlijk niet wil dan doe ik toch maar mee | ❑ | ❑ | ❑ | ❑ | ❑ |

BEWEGEN

## Vraag 13. Op hoeveel dagen per week ga je lopend of fietsend naar school of naar je stageplek?

❑ (Bijna) nooit

❑ 1 dag per week

❑ 2 dagen per week

❑ 3 dagen per week

❑ 4 dagen per week

❑ 5 dagen per week

## Vraag 14. Op hoeveel dagen per week doe je aan sport bij een club, vereniging of sportschool?

*Bijvoorbeeld: fitness, zwemmen, voetbal, tennis, dansen etc.*

❑ Ik sport niet bij een club, vereniging of sportschool

❑ Minder dan 1 dag per week

❑ 1 dag per week

❑ Meerdere dagen per week

❑ Elke dag

## Vraag 15. Op hoeveel dagen per week sport of beweeg je in je vrije tijd zonder club, vereniging of sportschool?

*Bijvoorbeeld: voetballen op straat, fietsen, hardlopen, sk eeleren, zwemmen, thuis dansen, kranten*

*bezorgen etc. (lopen en fietsen naar school niet meetellen).*

❑ (Bijna) nooit

❑ Minder dan 1 dag per week

❑ 1 dag per week

❑ Meerdere dagen per week

❑ Elke dag

We willen graag weten op hoeveel dagen per week je minstens 1 uur sport of beweegt. Tel alle vormen van sporten of bewegen die je op een dag doet bij elkaar op.

Bijvoorbeeld: op maandag fiets je 15 minuten naar school, je hebt 50 minuten schoolgym, je fietst 15 minuten terug naar huis en ’s avonds ga je 20 minuten hardlopen: op die dag heb je minstens 1 uur bewogen.

Doe dit voor alle dagen per week.

## Vraag 16. Op hoeveel dagen per week sport of beweeg je minstens 1 uur?

❑ (Bijna) nooit

❑ 1 dag per week

❑ 2 dagen per week

❑ 3 dagen per week

❑ 4 dagen per week

❑ 5 dagen per week

❑ 6 dagen per week

❑ Elke dag VOEDING

## Vraag 17. Op hoeveel dagen per week ontbijt je?

❑ (Bijna) nooit

❑ 1 dag per week

❑ 2 dagen per week

❑ 3 dagen per week

❑ 4 dagen per week

❑ 5 dagen per week

❑ 6 dagen per week

❑ Elke dag

## Vraag 18. Op hoeveel dagen per week eet je fruit?

❑ (Bijna) nooit

❑ 1 dag per week

❑ 2 dagen per week

❑ 3 dagen per week

❑ 4 dagen per week

❑ 5 dagen per week

❑ 6 dagen per week

❑ Elke dag

## Vraag 19. Op hoeveel dagen per week eet je groente?

❑ (Bijna) nooit

❑ 1 dag per week

❑ 2 dagen per week

❑ 3 dagen per week

❑ 4 dagen per week

❑ 5 dagen per week

❑ 6 dagen per week

❑ Elke dag ROKEN

De volgende vragen gaan over roken. Hiermee bedoelen we sigaretten en sjek, geen elektronische sigaret.

## Vraag 20. Heb je ooit gerookt?

❑ Ja, een hele sigaret of meer

❑ Ja, alleen een paar trekjes

## ❑ Nee Ga naar vraag 22 Vraag 21. Hoe vaak rook je nu?

❑ Elke dag

❑ Minstens 1 keer per week, maar niet elke dag

❑ Minder dan 1 keer per week

❑ Ik rook niet ALCOHOLGEBRUIK

## Vraag 22. Heb je ooit alcohol gedronken?

❑ Ja, een heel glas of meer

❑ Ja, alleen een paar slokjes

❑ Nee **Ga naar vraag 26**

## Vraag 23. Op hoeveel dagen heb je alcohol gedronken?

*Kruis op iedere regel één vak je aan.*

|  | Nooit | 1 of 2 dagen | 3 t/m 5 dagen | 6 t/m 9 dagen | 10 t/m  19  dagen | 20 t/m  29  dagen | 30  dagen of meer |
| --- | --- | --- | --- | --- | --- | --- | --- |
| In je hele leven | ❑ | ❑ | ❑ | ❑ | ❑ | ❑ | ❑ |
| In de laatste 4 weken | ❑ | ❑ | ❑ | ❑ | ❑ | ❑ | ❑ |

## Vraag 24. Hoe vaak heb je in de laatste 4 weken VIJF OF MEER drankjes met alcohol gedronken bij één gelegenheid (bijvoorbeeld op een feestje of op één avond)?

❑ Nooit

❑ 1 keer

❑ 2 keer

❑ 3 of 4 keer

❑ 5 of 6 keer

❑ 7 of 8 keer

❑ 9 keer of vaker

## Vraag 25. Hoe vaak ben je dronken of aangeschoten geweest door het drinken van alcohol?

*Kruis op iedere regel één vak je aan.*

|  | Nooit | 1 keer | 2 keer | 3 keer | 4 t/m 10 keer | 11 keer of vaker |
| --- | --- | --- | --- | --- | --- | --- |
| In je hele leven | ❑ | ❑ | ❑ | ❑ | ❑ | ❑ |
| In de laatste 4 weken | ❑ | ❑ | ❑ | ❑ | ❑ | ❑ |

MIDDELENGEBRUIK

## Vraag 26. Heb je ooit wiet (marihuana) of hasj gebruikt?

❑ Ja

❑ Nee **Ga naar vraag 28**

## Vraag 27. Op hoeveel dagen heb je wiet (marihuana) of hasj gebruikt?

*Kruis op iedere regel één vak je aan.*

|  | Nooit | 1 of 2 dagen | 3 t/m 5 dagen | 6 t/m 9 dagen | 10 t/m  19  dagen | 20 t/m  29  dagen | 30  dagen of meer |
| --- | --- | --- | --- | --- | --- | --- | --- |
| In je hele leven | ❑ | ❑ | ❑ | ❑ | ❑ | ❑ | ❑ |
| In de laatste 4 weken | ❑ | ❑ | ❑ | ❑ | ❑ | ❑ | ❑ |

## Vraag 28. Heb je ooit één van de volgende middelen gebruikt: XTC (ecstasy, MDMA), cocaïne, paddo’s, amfetamine (uppers, pep of speed), LSD, GHB, heroïne en/of lachgas?

❑ Ja

❑ Nee **Ga naar vraag 30**

## Vraag 29. Welke van de volgende middelen heb je gebruikt?

*Kruis op iedere regel één vak je aan.*

|  | Niet gebruikt | In de laatste  4 weken gebruikt | Wel gebruikt, maar langer dan 4 weken geleden |
| --- | --- | --- | --- |
| XTC (ecstasy, MDMA) | ❑ | ❑ | ❑ |
| Cocaïne (coke of wit) | ❑ | ❑ | ❑ |
| Paddo’s (hallucinogene paddenstoeltjes of magic mushrooms) | ❑ | ❑ | ❑ |
| Amfetamine (uppers, pep of speed) | ❑ | ❑ | ❑ |
| LSD | ❑ | ❑ | ❑ |
| GHB | ❑ | ❑ | ❑ |
| Heroïne | ❑ | ❑ | ❑ |
| Lachgas | ❑ | ❑ | ❑ |

# SCHOOL

**Vraag 30. Hoeveel dagen ben je de laatste 4 weken dat er school was thuis gebleven, omdat je ziek was?** *Vak antiewek en niet meetellen bij die 4 wek en.*

schooldagen

## Vraag 31. Hoeveel lesuren heb je de laatste 4 weken dat er school was gespijbeld?

*Vak antiewek en niet meetellen bij die 4 wek en.*

lesuren

PESTEN

De volgende vragen gaan over pesten. Pesten is bijvoorbeeld schelden, roddelen, vervelende berichtjes sturen, iets afpakken, spugen of iemand buitensluiten. Met pesten wordt hier bedoeld dat een persoon door anderen wordt getreiterd op een manier die deze persoon echt niet leuk vindt.

## Vraag 32. Hoe vaak ben je de laatste 3 maanden gepest op school?

❑ Nooit

❑ Minder dan 2 keer per maand

❑ 2 of 3 keer per maand

❑ Ongeveer 1 keer per week

❑ Meerdere keren per week

## Vraag 33. Hoe vaak heb je de laatste 3 maanden zelf een andere leerling op school gepest?

❑ Nooit

❑ Minder dan 2 keer per maand

❑ 2 of 3 keer per maand

❑ Ongeveer 1 keer per week

❑ Meerdere keren per week

De volgende vragen gaan over pesten via internet of mobiele telefoon. Het gaat om pesten op een manier die de ander echt niet leuk vindt. Voorbeelden zijn:

- iemand uitschelden via de telefoon
- iemand bedreigen, bijvoorbeeld in een tweet
- gemene roddels over iemand verspreiden op internet
- vervelende foto’s of filmpjes van iemand op internet zetten, bijvoorbeeld op YouTube

## Vraag 34. Hoe vaak ben je de laatste 3 maanden gepest via internet of mobiele telefoon?

❑ Nooit

❑ Minder dan 2 keer per maand

❑ 2 of 3 keer per maand

❑ Ongeveer 1 keer per week

❑ Meerdere keren per week

## Vraag 35. Hoe vaak heb je de laatste 3 maanden zelf een ander gepest via internet of mobiele telefoon?

❑ Nooit

❑ Minder dan 2 keer per maand

❑ 2 of 3 keer per maand

❑ Ongeveer 1 keer per week

❑ Meerdere keren per week

SOCIALE MEDIA

De volgende vraag gaat over sociale media. Met sociale media bedoelen we bijvoorbeeld:

- Berichten via smartphone, tablet, laptop of pc (zoals Whatsapp, Snapchat)
- Sociale netwerken (zoals Facebook, Twitter)
- Forums (zoals Fok.nl, Tweakers.net)

## Vraag 36. Hoe vaak ben je actief op sociale media?

❑ (Bijna) nooit **Ga naar vraag 38**

❑ Minder dan 1 dag per week

❑ 1 dag per week

❑ 2 of 3 dagen per week

❑ 4 of 5 dagen per week

❑ (Bijna) elke dag

| De volgende vraag gaat over jouw ervaring met sociale media.  **Vraag 37. Kun je van de onderstaande ervaringen aangeven hoe vaak je deze hebt?**  *Kruis op iedere regel één vakje aan.* | | | | | |
| --- | --- | --- | --- | --- | --- |
|  | Nooit | Zelden | Soms | Vaak | Zeer vaak |
| Hoe vaak vind je het moeilijk om met sociale media te stoppen? | ❑ | ❑ | ❑ | ❑ | ❑ |
| Hoe vaak zeggen anderen (bijvoorbeeld ouders of vrienden) dat je minder tijd zou moeten besteden aan sociale media? | ❑ | ❑ | ❑ | ❑ | ❑ |
| Hoe vaak gebruik je liever sociale media dan dat je in het echt tijd met anderen doorbrengt (bijvoorbeeld vrienden of ouders)? | ❑ | ❑ | ❑ | ❑ | ❑ |
| Hoe vaak voel je je onrustig, gestrest of geïrriteerd wanneer je geen sociale media kunt gebruiken? | ❑ | ❑ | ❑ | ❑ | ❑ |
| Hoe vaak raffel je je huiswerk af om sociale media te gebruiken? | ❑ | ❑ | ❑ | ❑ | ❑ |
| Hoe vaak ga je sociale media gebruiken omdat je je rot voelt? | ❑ | ❑ | ❑ | ❑ | ❑ |
| Hoe vaak kom je slaap te kort door sociale media? | ❑ | ❑ | ❑ | ❑ | ❑ |

# GAMEN

De volgende vraag gaat over gamen. Met games bedoelen we alle spellen/spelletjes die je speelt op een smartphone, tablet, laptop, pc, Mac of spelcomputer (zoals Playstation, Wii, Xbox).

## Vraag 38. Hoe vaak speel je games?

❑ (Bijna) nooit **Ga naar vraag 40**

❑ Minder dan 1 dag per week

❑ 1 dag per week

❑ 2 of 3 dagen per week

❑ 4 of 5 dagen per week

❑ (Bijna) elke dag

| De volgende vraag gaat over jouw ervaring met gamen.  **Vraag 39. Kun je van de onderstaande ervaringen aangeven hoe vaak je deze hebt?**  *Kruis op iedere regel één vakje aan.* | | | | | |
| --- | --- | --- | --- | --- | --- |
|  | Nooit | Zelden | Soms | Vaak | Zeer vaak |
| Hoe vaak vind je het moeilijk om met gamen te stoppen? | ❑ | ❑ | ❑ | ❑ | ❑ |
| Hoe vaak zeggen anderen (bijvoorbeeld ouders of vrienden) dat je minder tijd zou moeten besteden aan gamen? | ❑ | ❑ | ❑ | ❑ | ❑ |
| Hoe vaak ga je liever gamen dan dat je in het echt tijd met anderen doorbrengt (bijvoorbeeld vrienden of ouders)? | ❑ | ❑ | ❑ | ❑ | ❑ |
| Hoe vaak voel je je onrustig, gestrest of geïrriteerd wanneer je niet kunt gamen? | ❑ | ❑ | ❑ | ❑ | ❑ |
| Hoe vaak raffel je je huiswerk af om te gaan gamen? | ❑ | ❑ | ❑ | ❑ | ❑ |
| Hoe vaak ga je gamen omdat je je rot voelt? | ❑ | ❑ | ❑ | ❑ | ❑ |
| Hoe vaak kom je slaap te kort door gamen? | ❑ | ❑ | ❑ | ❑ | ❑ |

# SEKSUALITEIT

## Vraag 40. Heb je ooit met iemand gezoend (tongzoenen)?

❑ Ja

❑ Nee, nooit **Ga naar vraag 43**

## Vraag 41. Heb je ooit geslachtsgemeenschap gehad?

POP UP: *Sommigen noemen dit ‘sek s hebben’ of ‘neuk en’.*

❑ Ja, regelmatig

❑ Ja, een paar keer

❑ Ja, 1 keer

❑ Nee, nooit **Ga naar vraag 43**

## Vraag 42. Heb je altijd een condoom gebruikt toen je geslachtsgemeenschap had?

❑ Ja, altijd

❑ Nee, niet altijd

❑ Nee, nooit

❑ Ik weet het niet meer

# Dit is het einde van de vragenlijst.

## Vraag 43. Hoe heb je deze vragenlijst ingevuld?

*Je mag meer dan één antwoord geven.*

❑ Op een pc of laptop

❑ Op een tablet

❑ Op een smartphone

❑ Op papier

**Bedankt** voor het invullen van de vragenlijst!
